# ‘Pawing’ uncertainty! how dogs attenuate the impact of daily hassles at work on uncertainty

**DOI:** 10.1186/s40359-023-01295-z

**Published:** 2023-08-29

**Authors:** Ana Junça-Silva

**Affiliations:** 1 Instituto Universitário de Lisboa (ISCTE-IUL), Lisboa, Portugal; 2 Business Research Unit – BRU (UNIDE-IUL), Avenida das Forças Armadas, 1649-026 Lisboa, Lisboa, Portugal

**Keywords:** Daily hassles, Uncertainty, Adaptive performance, Dogs at work, Human-animal interactions, The health benefits of dogs

## Abstract

**Purpose:**

This study relied on the integrative model of uncertainty tolerance to delineate an argument proposing that daily hassles trigger uncertainty, and this influences adaptive performance. Furthermore, relying on the “*furr-recovery method”* –where interactions with dogs allow dog owners to recover from negative situations or job demands - this study tested whether having a dog would moderate the relationship between daily hassles and uncertainty.

**Methodology:**

To test this proposed model, daily data during ten working days was gathered with a sample of white-collar workers who were teleworking (*N* = 233 × 10 = 2,330).

**Findings:**

Multilevel results showed that daily hassles influenced adaptive performance via perceived uncertainty. However, the relationship between daily hassles and uncertainty was conditional on the ownership of a dog, in such a way that the relationship became weaker for those who had dogs. That is, those who did not have dogs had increased levels of uncertainty after daily hassles when compared to those who had dogs.

**Practical implications:**

Managers may consider the adoption of pet-friendly work practices (for instance, telework – working from home allow employees to work nearby and interact with their dogs during worktime) as dogs appear to have a beneficial effect to help employees effectively cope with daily hassles and reduce their uncertain reactions.

**Originality:**

This study advances knowledge regarding the *pawing-effect* (the reduced uncertainty to daily hassles on dog owners) on employees’ uncertainty to daily hassles and opens new venues for research regarding their role in work-related outcomes. Further, future research could examine how human-dog interactions or the quality of their relationship may benefit owners and explore the benefits of bringing dogs to work periodically.

## Introduction

*“The bond with a true dog is as lasting as the ties of this earth will ever be.”* (Konrad Lorenz, 1950).

The affective events theory (AET) [1] explains how and when daily micro-events influence affective states [2–4], cognitions [5, 6] and behaviors [7, 8]. Accordingly, the work context (telework or face-to-face) promotes conditions for the occurrence of different types of daily micro-events, such as daily hassles [9, 10]. Daily hassles are tiny occurrences that trigger irritation, frustration, or other negative states [3], and may include for instance having to deal with unexpected changes in the tasks to perform [4]. This kind of daily hassle will likely make employees experience uncertainty – a subjective experience of ignorance [5] – because they might not always know what to do [6]. When employees perceive uncertainty after the experience of daily hassles, the ability to adapt to unexpected and changing work situations (i.e., adaptive performance) will likely be impaired [7–9]. However, this depends on the context and the other variables that can influence this relationship, such as personality or context variables, such as the presence of a pet [8].

Relying on the “furr-recovery method” [11, 12], I argue that dog owners will react differently to daily hassles when compared to employees who do not own dogs. The furr-recovery method explains that interacting with a pet during work time will serve as a micro-break that restores lost energy, stamina, and motivation, making the individual ready to invest additional efforts in their work [11, 12]. Hence, based on this, we expect that those who own dogs will deal with daily hassles differently from those who do not own dogs. Dogs are a source of emotional support [13]; as such, they might create conditions for their owners to feel better even when they have a demanding day, with several daily hassles at work. Therefore, the relationship between daily hassles and perceived uncertainty will be weaker for dog owners when compared to those who do not own dogs.

The AET is a well-established theory and has been demonstrated across different working contexts and regarding different kinds of behaviors [e.g., 14]; however, this study goes further for two reasons. First, the adoption of perceived uncertainty as a potential mechanism in the relationship between daily hassles and adaptive performance is still to be unveiled; even though the path from uncertainty to adaptive performance has been well-determined, these findings are ambiguous as some of them show a positive path, and other a negative one [e.g., 8]. Second, pets at work are a hot topic in organizational psychology [15]; however, there is still much to learn about the role of dogs in the work context, namely whether they will be an important boundary condition able to mitigate the negative impact of daily hassles on their owners’ perceived uncertainty and their resultant adaptative performance.

Based on that, this study makes some theoretical and practical contributions. First, including the role of dogs as an individual difference in the AET will help to expand the theory by adding another condition that may amplify or mitigate the effects of daily micro-events [11]. Second, by doing so it may help managers to delineate strategies (for instance, implementing pet-friendly practices) that help their employees to better deal with daily hassles that may trigger uncertainty.

## Theoretical framework

### The affective events theory

Daily micro-events are a constant in daily life at work. The AET has emphasized the role of daily micro-events for diverse work-related outcomes, such as performance [1]; accordingly, the work environment and work routines create conditions for these events to occur [1]. Further, when daily micro-events occur, the employees’ affect, cognitions, and behaviors are influenced [e.g., 10].

Daily micro-events may be positive (daily uplifts) or negative (daily hassles). While daily uplifts are micro-positive experiences that tend to boost daily satisfaction [4], daily hassles are the tiny things that upset employees [14] and may create negative reactions that may range from feeling frustrated to appraising work-related uncertainty [e.g., 16]. Some examples of daily hassles may include issues related to the technology needed to perform the tasks (e.g., slow computer or slow network connection), or having to deal with someone in a rotten mood. Regardless of the reaction, daily hassles may harm employees’ performance levels due to the reactions triggered by such daily micro-events [16–18].

## The mediating role of perceived uncertainty

The path from daily hassles to uncertainty and from this to performance can be explained by the integrative model of uncertainty tolerance (IMUT) [8]. Accordingly, daily events appraised as unknown are stimuli that trigger the perception of uncertainty; this, in turn, leads to cognitive (e.g., appraisals of denial, vulnerability, doubt, or threat), affective (emotions, such as worry, fear, disinterest) or behavioral (e.g., avoidance, inaction or approach) responses [19; 20]. The model also states that there may exist individual differences (e.g., personality or variables of the context, such as the presence of a pet) that may buffer or intensify the levels of the perceived uncertainty to the stimuli experienced (that is, daily hassles or other negative events).

One of the employee’s biggest challenges in daily life at work is coping with the various events that account for perceived uncertainty [21, 22]. There are different definitions of perceived uncertainty, that range from the possibility that daily hassles - harmful events - may occur [19, 23], to the period of anticipation before such daily hassles [24], to the “notion that negative events may occur and there is no definitive way of predicting such events” [25, p. 106]. Therefore, perceived uncertainty may be defined as a mental state, a subjective and cognitive experience of ignorance – i.e., the lack of knowledge or the so-called conscious awareness of what surrounds the individual [25]. Furthermore, uncertainty is an aversive and alarming experience [26] with consequences for employees’ behaviors, as it may make them worry about the control they have over their work-life [21].

When daily hassles lead employees to feel uncertain (like changing the work procedures or routines), they may respond negatively against the organization, decreasing their performance [27, 28]. Lind and Van den Bos [21] argued that daily hassles are particularly threatening in the face of great uncertainty and drive people to perceive even more uncertainty and act against the organization because “harming the organization is as much as a goal as protecting the self” (p. 196). As such, by creating the perception of uncertainty, daily hassles may reduce the ability of individuals to adapt to changing conditions and unexpected events.

The way in which employees deal with uncertainty may differ at the between and within-person level; that is, on some occasions employees may adapt to uncertain conditions, and on other occasions they can exhibit avoidance responses [8]. Further, even between individuals there may exist different responses depending on individual or contextual factors (such as, having a dog nearby may help individuals to better cope with negative or uncertain events). Even the findings may seem inconsistent because some studies reported positive outcomes and other reported negative ones; for instance, some studies have shown that uncertain events and unexpected occurrences may provide the opportunity for individuals to adapt and improve their ability to respond positively [8].

The ability to adapt or adaptive performance is a facet of job performance and emerged from the increasingly volatile, uncertain, complex, and ambiguous (VUCA) world of work [29]. Furthermore, the increasing changes in the work environment and its impact on the nature of work emphasized the need for adaptive behaviors from employees – that is, their adaptive performance [30–32]. Employees show adaptive performance when they are able to adjust their behaviors to the requirements and demands of daily hassles or new work situations [30]. Thus, adaptive performance is the employee’s ability to adapt to dynamic, uncertain and unexpected occurrences, such as daily hassles [9].

Based on the AET and the IMUT, it is expected that daily hassles will likely increase employees’ perceived uncertainty which, in turn, will influence their adaptive performance.

### Hypothesis 1

Daily uncertainty will mediate the relationship between daily hassles and daily adaptive performance, at the within-person level.

### The moderating role of dogs

Uncertainty threatens one’s general sense of self and may decrease performance [33], thus people try to find some way to tolerate it or to make it more manageable [22]. The IMUT [8] argues that there are factors that can buffer or amplify the individuals’ uncertainty to daily hassles.

Dogs – or a human’s best friend – create deep bonds with their humans [34] and are a source of emotional support for them [35]. Furthermore, the therapeutic role of dogs in stress or anxiety relief has been consistently demonstrated [e.g., 36; 37], as well as their role in boosting humans’ well-being and happiness [e.g., 38]. Indeed, some studies have demonstrated that human-animal interactions reduced the levels of cortisol, a stress hormone, and heart rate which contributed to relaxing individuals [e.g., 39].

Recently, Junça-Silva [11, 12] demonstrated that human-animal interactions in the work context not only relaxed employees but created moments for them to recover and respite – the *furr-recovery* method. Accordingly, interacting with pets (e.g., petting the dog’s head) during work time may be a micro-break that distracts employees from work and helps them recover self-regulatory resources that are needed for them to deal with daily hassles. Hence, having a pet nearby may help employees to feel better by reducing their stress and anxiety levels and improving their affective and mental well-being [11, 12]. Plus, interacting with a dog during a workday may provide stability that could reduce anxiety even when daily hassle occurs [40]. As explained earlier, distractions from work or micro-breaks (such as, stop working to interact with the dog) are relevant conditions to minimize the detrimental effects of negative events or daily hassles on perceived uncertainty [12]. Micro-breaks that include human-dogs interactions help to recover energy and self-regulatory resources needed to regulate emotions and negative thoughts that are inherent to uncertainty [40]. Hence, dogs may be a boundary condition that alleviates the levels of perceived uncertainty after daily hassles.

The role of dogs in working settings is a hot topic and answers the call for studies by Kelemen et al. [15]. There are some empirical demonstrations of their benefits for personal and work-related outcomes. For instance, Junça-Silva [11] demonstrated that human-animal interactions at work boosted employees’ self-regulatory resources that, in turn, increased their levels of both task and adaptive performance. Similarly, Junça-Silva [12] showed, in a diary study, that having a pet nearby not only promoted positive affect but also contributed to employees’ mental health at the end of the day. Furthermore, Junça-Silva [40] also evidenced that human-animal interactions at work prompted positive affective experiences more regularly and these accounted for increases in work engagement. Wagner and Pina-Cunha [34] showed that dogs at the workplace had a positive influence on individual and collective well-being of organizational members in an office environment. Similarly, Pina-Cunha et al. [41] evidenced that dogs were important at the workplace due to the positive effects of their presence on workers’ attitudes and behaviors. Therefore, pets at work, despite the scarce number of studies [42], appear to be a boundary condition that may amplify positive responses or buffer the negative ones after daily hassles.

Therefore, relying on the *furr-recovery* method, it is expected that those who have dogs have resources that help them to better manage their perceived uncertainty to daily hassles.

#### Hypothesis 2

**Fig. 1:**
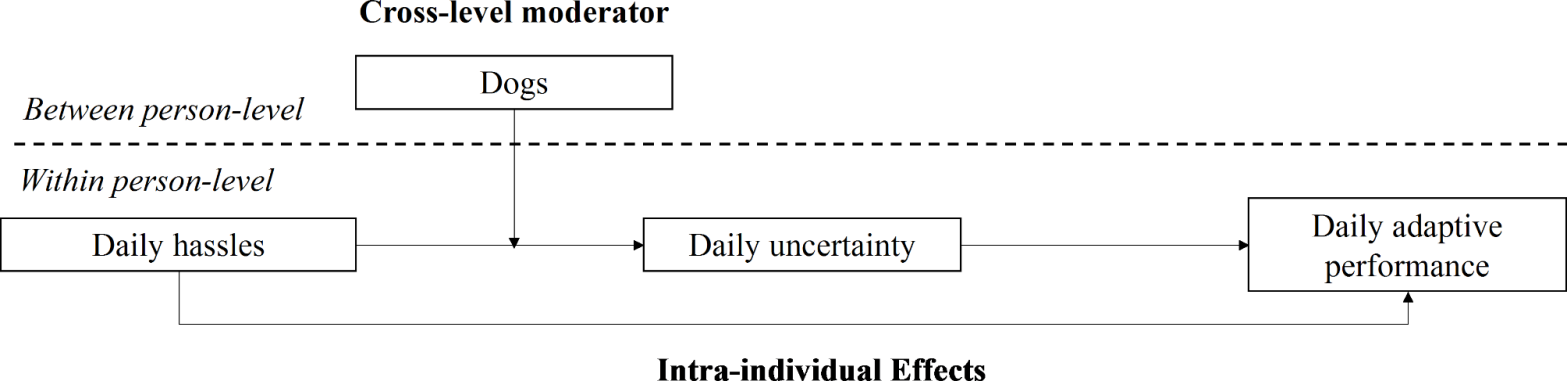
Multilevel proposed model

Dogs moderate the positive within-person relationship between daily hassles and daily perceived uncertainty such that for those who have dogs (vs. those who do not have), daily hassles will be weakly related to perceived uncertainty (see Fig. 1).

## Method

### Procedure

This study resorted to multilevel research that included one general and daily diary surveys answered for 10 workdays (from Monday to Friday for two weeks). All the scales were presented in Portuguese after a process of translation and back-translation. All the surveys (general and daily) were answered online as a way to check the date and time on which respondents answered them. Participants were reminded every day, at the end of the working day, to answer the daily survey (they had to answer by 11 p.m. of that day). Data were collected between October and November 2022.

Overall, 300 Portuguese working adults, from the researcher’s professional networks (LinkedIn), were asked to take part in this study, of which 286 completed the general survey (response rate: 95.3%), 251 completed at least one diary survey (response rate: 83.6%), and 233 completed all 10 daily online surveys (response rate: 77.6%, measurement occasions = 2,330). This sample size is considered adequate because as suggested by Maas and Hox [43] when the aim is to perform cross-level interactions (i.e., between-person moderators on a within-person relationship), level-2 variables (i.e., dogs) must exceed 30 respondents in a multilevel framework (diary nested in persons) to produce an accurate estimation of standard errors. Thus, a sample of 233 participants had satisfactory power and accuracy, as it exceeded the minimum sample requirements [43].

### Participants

Overall, 49.3% had dogs and 50.7% did not; mostly they were female (60%), 60% held a university degree and 40% held a high school diploma. Participants were on average 33.71 years old (*SD* = 12.72) and their mean organizational tenure was 13.40 years (*SD* = 4.57). They reported working on average 35.48 h per week (*SD* = 13.77). They worked in diverse occupational sectors, including management (42.5%), services (35.2%), and education (22.3%). Furthermore, participants were teleworking in a hybrid format (80.7%) or fully teleworking (19.3%). On average, participants who had dogs indicated having three dogs (*SD* = 4), and they had them at least for 12 years (*SD* = 9.21).

### Measures

#### Screening survey

A general survey was used to collect sociodemographic data (i.e., gender, age, tenure, and educational level) and the between-person variable – information relative to their dog ownership. Participants were asked whether they had (or not) a dog(s). They answered using a dichotomic response (1-*no*, 2 – *yes*).

### Daily survey

The recommendations procedure for daily diary methods were followed [e.g., 44]. As such, to reinforce the daily nature of the survey, all items were worded such that they included “today,” and used the past tense in each item. Moreover, to improve reliability and lessen participants’ drop-out rate, short scales were used. Finally, the level-specific composite reliability (i.e., within-person ω) was tested, as Geldhof and colleagues [45] suggested.

**Daily hassles.** The scale for daily hassles and uplifts at work was used to measure the frequency of daily hassles (SDHUW) [46]. It included 11 items that measure daily hassles (e.g., “Today, at work, I had to deal with someone in a rotten mood.”) and were answered on a five-point scale (1-*never;* 5 – *four times or more*). The within-person omega reliability coefficient was good (ω = 0.79).

**Perceived uncertainty.** Three items from the Organizational Change Scale [47] were used. An example item is: “Today, I was unsure about how to react to changes”. All items were answered on a five-point scale (1 = *strongly disagree*, 5 = *strongly agree*). (ω = 0.82).

**Adaptive performance**. Performance was measured through the 3-item of the Individual Task Adaptivity Scale of Griffin et al. [48]. Respondents rated the items on a 5-point Likert-type scale ranging from 1 (*very little*) to 5 (*a great deal*). An example item is: “Today, I adapted well to changes in core tasks” (ω = 0.79).

**Control variables.** The time of data collection (from Monday to Friday – a within-person variable that ranged from 1 to 10 – e.g., “1” for the first Monday and “10” for the second Friday) was used as Level 1 control variable, because due to the daily diary nature of the data, it could influence the criterion variables and produce memory or leaning bias [e.g., 49].

### Data analysis

JASP was used to perform confirmatory factor analyses (CFA) and SPSS with the macro-Multivel mediation (MlMed) to assess the proposed multilevel model [50]. This macro was used because it is particularly relevant when testing 1-1-1 multilevel mediations, and cross-level interactions [50]. First, Mlmed is useful as it tests the 1-1-1 indirect effect with Monte Carlo simulations generating 95% CI using 10,000 resamples; this is relevant to minimize the potential bias in multilevel mediation estimates [51]. Second, the macro estimates both within-person and between-person variables. To estimate within-person effects, Mlmed person-mean centers variables by subtracting the participants’ general mean from their mean reported for each day. The within-person effects specify the extent to which participants’ person-centered score of an independent variable is related to their person-centered score of another variable (e.g., daily hassles and daily uncertainty). Furthermore, between-person effects provide evidence of Level 2 relationships. To estimate between-person effects, Mlmed enters person-means at Level 2 (e.g., the mean of a variable across the 10 days). Hence, the between-person effects specify the extent to which an individual mean across the 10 days deviates from the grand mean (i.e., mean across all participants in the study).

As the study had a multilevel data structure, that is days nested in individuals, and to justify using a multilevel modeling approach, I started to estimate the intra-class correlation coefficient (ICC) for daily hassles, perceived uncertainty, and adaptive performance [52]. The results indicated that a significant proportion of the variance (ICC values were 0.45, 0.43, and 0.34, respectively) was attributable to within-person fluctuations. Between-person variance represents the relative differences among participants’ overall variable levels whereas within-person differences represent a participant’s change in a particular variable from one day to the next [53]. Moreover, because all the ICCs were higher than 0.05, it could be concluded that the data had indeed a multilevel structure (days nested in individuals) [54]. As such, based on the reported ICCs following a multilevel modeling approach appears to be a valid strategy [55].

CFAs were tested in JASP to understand whether there were different measures of the constructs and to understand the presence of the potential common method bias in the results. Table 1 presents the fit statistics. The first measurement model (M1) is the hypothesized model, including the following three latent factors: daily hassles, perceived uncertainty, and adaptive performance. Three alternative CFA models have been tested: (1) one alternative model comprised two latent factors in which daily hassles and perceived uncertainty were loaded onto one factor (M2). At last, a CFA with only one latent variable was tested (M3) – that is, all the variables were loaded onto one factor. Therefore, the model fit for each of these CFAs was evaluated and compared. The models were evaluated based on the root mean square error of approximation (RMSEA), the comparative fit index (CFI), the Tucker–Lewis index (TLI), and the standardized root mean square residual (SRMR). As Schreiber et al. [56] described, a model presents a good fit when the values of both CFI and TLI are higher than 0.90’s; when the values of both RMSEA and SRMR are below 0.08. Following these criteria, the hypothesized measurement model (M1) had an acceptable fit with the data. In addition, all the models were compared to the proposed one (M1) through a χ^2^-difference test. The χ^2^-difference test indicated that the hypothesized model presented the best fit to the data. Hence, the hypothesized model was the one with the best fit for the data. Further, together with the reliability of each variable, it could be assumed that these results evidenced that the variables were distinct constructs at the within-person daily-level and that the common method bias was not a severe issue in this study.

**Table 1:** *Comparison of measurement models*

Model		*χ2 (df)*	RMSEA	CFI	TLI	SRMR_within_	SRMR_between_	Comparison	Δ*χ2*	Δ*df*	*P*
M1	*3 latent factors*	*642.201 (62)*	*0.07*	*0.98*	*0.97*	*0.06*	*0.06*	*-*	*-*	*-*	-
M2	2 latent factors	2648.474 (64)	0.16	0.91	0.89	0.13	0.12	M2-M1	2006.273	2	*<* 0.001
M4	1 latent factor	9589.365 (65)	0.30	0.66	0.59	0.20	0.20	M3-M1	8947.164	3	*<* 0.001

*Note.* RMSEA: root mean square error of approximation; CFI: comparative fit index; TLI: Tucker–Lewis’s index; SRMR: standardized root mean square residual;

Best-fitting model in italics

M1: daily hassles, perceived uncertainty and adaptive performance fit load onto three separate latent factors

M2: daily hassles and perceived uncertainty were loaded onto one latent factor plus adaptive performance was loaded onto one separate latent factor

M3: all the variables (daily hassles, perceived uncertainty and adaptive performance) were loaded onto one single factor

## Results

### Descriptive results

Table 2 presents the descriptive statistics and zero-order and person-centered correlations of the variables to be tested.

**Table 2:** Means, standard deviations, and between-and within-person level correlations

Variables	*M*	*SD*	1	2	3	4	5
1. Daily hassles	1.96	0.77	-	0.27**	-0.10*	-0.11*	-0.09*
2. Perceived uncertainty	2.82	0.73	0.32**	-	0.23**	-0.06*	0.02
3. Adaptive performance	3.42	0.83	-0.19**	0.03	-	0.08*	-0.08*
4. Dogs	-	-	0.02	-0.06*	0.15**	-	0.06*
5. Time	-	-	0.02	0.03	0.04	0.01	-

*Note*. Correlations below the diagonal are between-person level. Correlations above the diagonal are within-person level. Time variable ranged between 1 to 10 (the number of days of the data collection). *N*_(observations)_ = 2,330; *n*_(participants)_ = 233. Dogs: 1-no; 2-yes. ***p < 0.001, ** *p* < 0.01, *p < 0.05

**Fig. 2:**
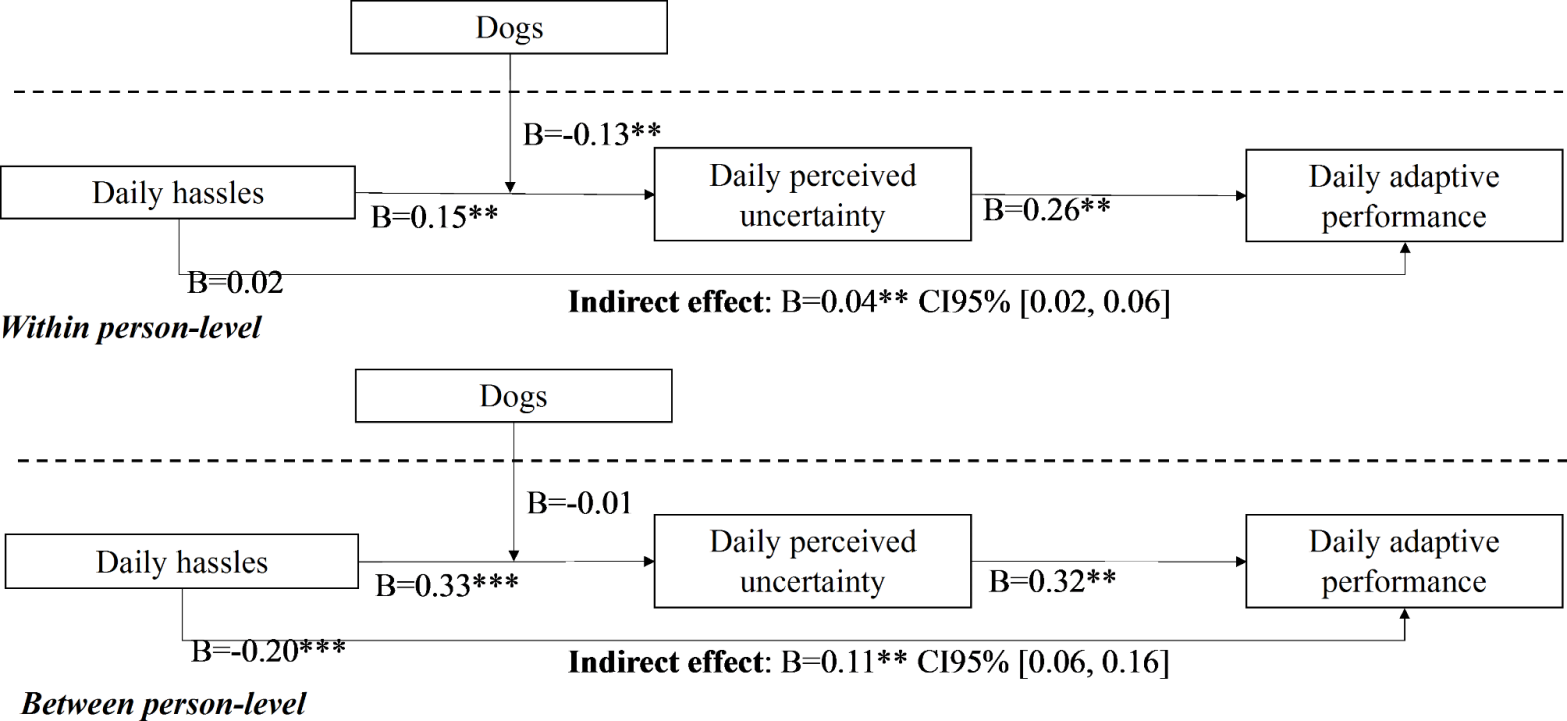
Summary of multilevel path estimates both at the within and between-person level

#### Means comparison between groups

Before testing the hypotheses, differences in the variables under study between the two groups (dog owners and non-dog owners) were examined. Results showed statistically significant differences for daily hassles (*F*_(2328)_ = 6.931, *p <* 0.01), showing that dog owners reported fewer daily hassles (*M* = 1.92, *SD* = 0.74) than those without dogs (*M =* 2.05, *SD =* 0.82). Furthermore, the results also evidenced statistically significant differences for daily adaptive performance (*F*_(2328)_ = 41.121, *p* < 0.001), where participants with dogs scored higher on adaptive performance levels (*M* = 4.12, *SD* = 0.58) compared to those without dogs (*M* = 3.87, *SD* = 0.76). Despite the lower values of daily uncertainty reported by dog owners (*M* = 2.75, *SD* = 0.72) compared to non-dog owners (*M* = 2.84, *SD* = 0.77), these were not statistically significant (*F*_(2328)_ = 0.92, *p* > 0.05).

### Hypotheses testing

As suggested by Griep et al. [57] the models were analyzed regarding the one that best fitted the data. As such, the Bayesian information criterion (BIC) – that is the balance between the number of parameters (i.e., model complexity) and the fit of the model to the data – was analysed. The BIC and the sample size–adjusted BIC values were compared between the multilevel 1-1-1 mediating model with the cross-level moderation model. The findings showed that the multilevel cross-level moderation model was the one with the lowest BIC value, hence it was the one that presented the best fit to the data (BIC = 6357.05; sample size–adjusted BIC = 6361.05) when compared to the multilevel mediating model (BIC = 6579.70; sample size–adjusted BIC = 6547.54). Figure 2 presents the estimated paths of the model.

First, daily hassles (Estimate_within_=0.15, 95% CI = [0.09, 0.21]; Estimate_between_=0.33, 95% CI = [0.24, 0.42]) related positively to daily perceived uncertainty; and daily perceived uncertainty was positively related to daily adaptive performance (Estimate_within_ = 0.26, 95% CI = [0.19, 0.32]; Estimate_between_=0.32, 95% CI = [0.20, 0.44]). Moreover, the results showed a significant indirect effect from daily hassles to daily adaptive performance (Estimate_within_ = 0.04, 95% CI = [0.02, 0.06]; Estimate_between_=0.11, 95% CI = [0.06, 0.16]) via daily perceived uncertainty, thereby p is 1.

**Fig. 3:**
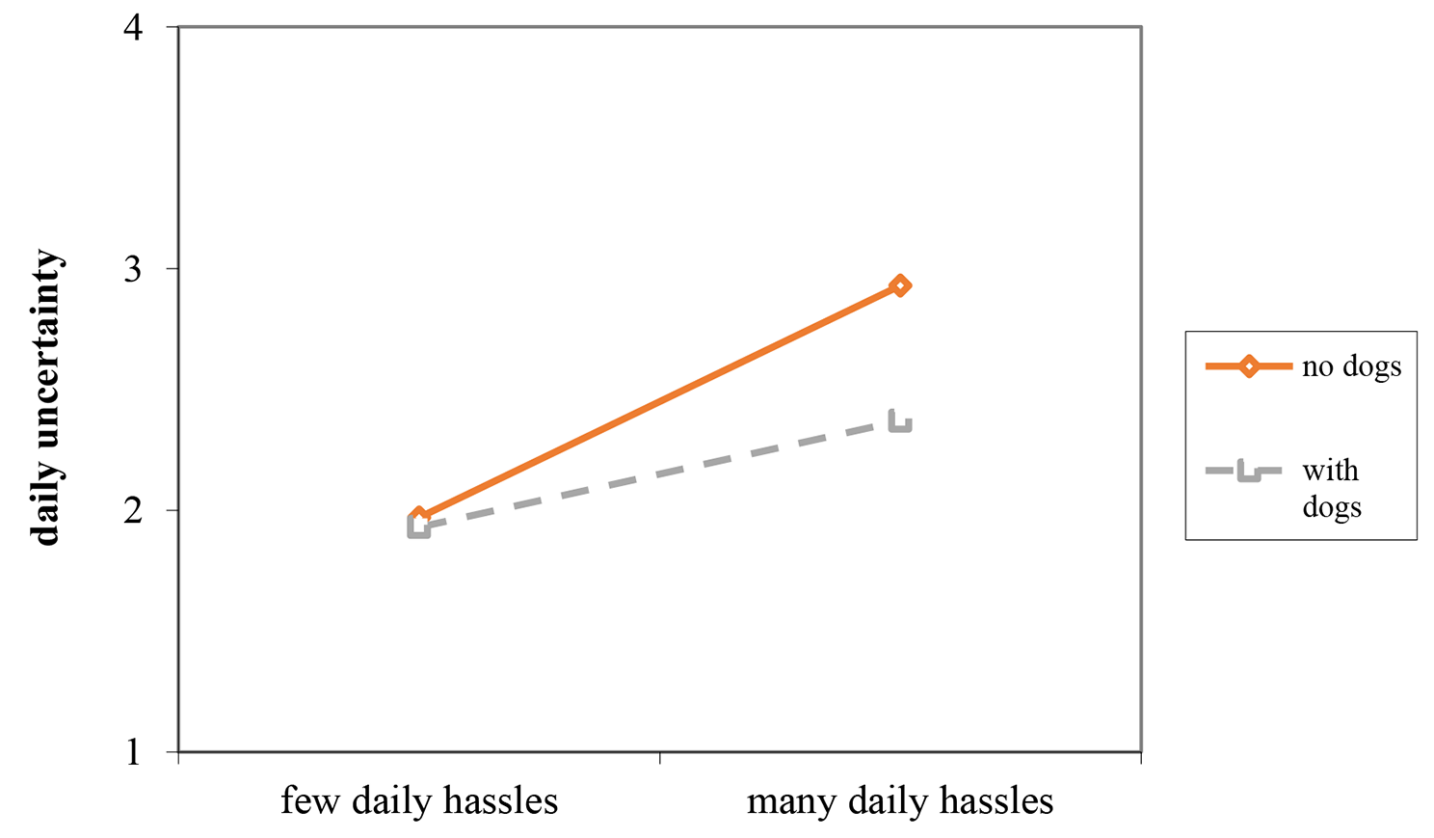
Interaction between daily hassles and having (or not) dogs

Next, hypothesis 2 was tested. The results showed a negative cross-level relationship between dogs and daily hassles regarding daily perceived uncertainty (Estimate_within_ = -0.13, 95% CI = [-0.26, -0.01]; Estimate_between_=-0.01, 95% CI = [-0.19, 0.19]). The results showed that dogs buffered the positive relationship between daily hassles and daily perceived uncertainty. As Fig. 3 shows, the strength of the relationship between daily hassles and daily perceived uncertainty was smaller for employees who owned dogs (B = 0.11, p > 0.05) in comparison to employees who did not own dogs (B = 0.240, p < 0.01). Put differently, daily perceived uncertainty was less dependent on daily hassles when employees had dogs on their own. Hypothesis 2 was thus supported.

## Discussion

This study aims to give a step forward in understanding the role of dogs in the work context, particularly regarding the relationship between daily hassles and perceived uncertainty. In a world increasingly uncertain, as it is the world of work [29], it is never too much to understand which strategies might help to reduce it. Hence, answering the call for studies on the intersection of pets with daily work life [see 15], this research aims to examine the role of dogs in the relationship between daily hassles and triggered uncertainty. It also aims to explore whether uncertainty may serve as a mechanism connecting daily hassles to adaptive performance.

Overall, this study demonstrates that daily hassles lead to increased levels of uncertainty and this, in turn, influences adaptive performance. Furthermore, having a dog appears to attenuate the relationship between daily hassles and uncertainty, as the levels of uncertainty appear to increase at a lower rate, compared to those who did not own dogs, after experiencing daily hassles.

### Theoretical implications

First, the results evidence that daily hassles – micro-events that upset employees - make employees perceive uncertainty [i.e., the conscious experience of ignorance; 58] and this, in turn, contributes to improving employees’ adaptive performance [their adaptability to the work dynamics of change; 30]. The IMUT [8] supports this evidence as it argues that there are stimuli (situations or events) that make employees perceive uncertainty. When this happens, i.e., when individuals perceive uncertainty, they might approach it (and engage in adaptive behaviors) or avoid it (and not act or lose focus) [8]. Hence, daily hassles, by triggering uncertainty reactions, may signal that some adaptability is needed to deal with such events. Furthermore, a balanced level of uncertainty might be a resource to identify when adaptability is needed.

Notwithstanding, the empirical studies have brought confusing and ambiguous results regarding the role of uncertainty on adaptive performance [59]; while some scholars argue that perceived uncertainty is bad for adaptive performance [e.g., 60], others emphasize its positive effect [8]. Perceived uncertainty, at a balanced level, may positively influence adaptability by creating eustress; whereas a high level of perceived uncertainty in the workplace can decrease adaptive performance because it may trigger higher distress levels that may impair efficient adaptation [16]. In this sense, the findings show a moderate level of perceived uncertainty at the within-person level (M = 2.82) which may explain why adaptive performance is improved and not decreased [as many studies have described; 59]. Hence, a moderate number of daily hassles may increase the perception of uncertainty (with moderate levels) which may indicate when adaptability is needed.

The results of this study demonstrated that the relationship between daily hassles and resultant perceived uncertainty is moderated by the presence of dogs. That is, employees who owned dogs had decreased levels of perceived uncertainty even when daily hassles were more frequent. Before discussing this result, it is important to emphasize that research has amply shown that the relationships between dog/pet ownership and physical and mental health are not clear-cut. There are studies that show that pet owners derive physical and mental health benefits from their pets [e.g., 61; 34; 42], but there are other studies showing no relationship or relationships in the opposite direction, where negative mental health conditions like depression and anxiety are higher in pet owners than non-owners [62]. This can also be explained by the fact that pet owners may feel stress and anxiety from the cumulative burden of pet care, especially if resources are limited [63]. While so far research has shown ambiguous results, this study evidences the benefits of having a dog as a relevant boundary condition regarding the impact of daily hassles on triggered uncertainty.

First, it is important to highlight that most employees in this research were white-collar workers (with superior hierarchical positions) and were mostly teleworking (fully or in a hybrid format). Thereby, these employees might benefit from the presence of their dog while working. Recent studies have shown that people often report that when they feel their stress level rising, they stop to interact with their pets as a way to seek emotional support and reduce their distress and anxiety levels [64; 65]. For instance, Queen Elizabeth II gave a real example of the benefits of dogs. She often sought comfort and emotional support from her Corgis as a way to deal with her anxieties, pressures, and kingdom concerns - close family members named this refuge: the dog’s mechanism. Recent systematic literature reviews showed the main benefits of dogs for individuals; for instance, Rodriguez et al. [66] showed, in their systematic review, that having an assistance dog had beneficial effects on psychological well-being, emotional functioning, self-esteem, and vitality. Similarly, a systematic review, developed by Gee and Mueller [65], evidenced that the most frequent benefits of having a pet were increased physical health and exercise together with improved social functioning and decreased levels of depression and anxiety, and loneliness. Furthermore, Lundqvist et al. [67] developed a systematic review of the effects of dog-assisted activities, therapy, and support. They found that dog-assisted therapy had benefits in the treatment of psychiatric disorders among both young and adult patients; dog-assisted activities had benefits on health, well-being, depression, and quality of life for patients with severe cognitive disorders, while dog-assisted support had positive effects on stress and mood. Hence, dogs appear to have benefits in some well-being-related indicators.

Additionally, some studies have shown that human-animal interactions (e.g., petting the dog’s head or belly) are micro-breaks that restore self-regulatory resources needed to workers perform their tasks [11, 12] – the *furr-recovery* method. Also, Teo and Thomas [68] found that pet owners who had secure pet attachments showed lower psychological distress and psychopathology, and higher quality of life. Hence, the findings expand the IMUT and show that dogs may have a role as a situational boundary condition that may minimize the role of certain stimuli (in this case, daily hassles) on perceived uncertainty.

It is important to emphasize that dogs appear to give some stability to their owners as there are no significant differences in the levels of uncertainty for those who own dogs; on the opposite, those who do not own dogs appear to be more vulnerable to such situational influences (i.e., daily hassles) and, as such, their levels of uncertainty are significantly higher at the end of a day filled with daily hassles.

Owning a dog may be more than emotional support, or a companion [64]. Dogs not only provide a source of emotional support [15], but they are also a company – the best company to face the mandatory lockdowns as shown by Bussolari et al. [71] and Morgan et al. [72] – and the stability that may be helpful to face daily hassles [69, 70]. Hence, dogs are the lifeboat for when people feel adrift, especially on days when there are frequent daily hassles and where volatility, uncertainty, complexity, and ambiguity seem to be the constant of the current world of work. In sum, there may be a *pawing-effect* (decreases in uncertainty after daily hassles for those who own dogs) regarding employees’ perceived uncertainty.

#### Practical implications

The results of this study are relevant for managerial purposes. First, managers may consider the implementation of pet-friendly policies in their organizations. For instance, the adoption of telework, even in a hybrid regimen, may be suitable, particularly for those who own dogs and who cannot take them to the organization’s facilities [11, 34].

Organizations could also implement “dog day at work”, in which employees would be allowed to take their furry friends to work with them on that day. Lastly, the implementation of this day for longer periods, by creating appropriate facilities appears to be a positive mechanism through which employees excel in their performances and boost their happiness [34] (see Amazon as an example). It is relevant to emphasize that this strategy should be well thought out and structured before its implementation because (1) not all dogs are suited to work environments (for instance, dogs who need more attention, dogs with extraverted and strong personalities, or those who are extremely energetic) as they can cause additional work-related stressors [34], (2) there are also physical health concerns that must be considered (e.g., workers with allergies, phobias, religious matters, or those who purely do not like dogs), (3) and this could also compromise dog welfare as it could be a source of anxiety and distress for him/her [61].

### Limitations and future directions

Despite the positive contributions of this study, there are some considerations to bear in mind. First, the self-reported nature of the data may have caused the common method bias [73]; however, daily hassles and perceived uncertainty – that is, daily micro-events and an inner cognitive state – are measured in a better way through self-reported measures, because the individual is the one who best knows what happens to him/her and how s/he feels about it [74].

Second, I only focused on the negative nature of daily micro-events, but future studies would test the model assessing daily uplifts. Furthermore, this study only assessed the adaptive component of job performance; hence, future research should analyze the model regarding the other dimensions (i.e., task and contextual performance, and counterproductive work behaviors).

Third, the sampling method resorted to a non-probabilistic convenience sample which may give rise to sampling bias reported, such as the high proportion of females. For instance, women tend to report higher attachment to pets when compared to men [e.g., 75]. This may have contributed to the influence of the present findings. For that reason, other studies should replicate this model with a more balanced sample (the same proportion of both males and females).

Fourth, this study only considers the role of dogs; however other pets may have similar effects. For instance, cats can also be a significant source of emotional support and relaxation for their owners, and as such, cats can also moderate the relationship between daily hassles and perceived uncertainty. Hence, future studies should consider the inclusion of cats to test this or similar models.

At last, the study only considered dog ownership as a moderator in the conceptual model. However, as emphasized earlier, interactions with dogs can provide a palliative effect [61; 64]. There can be other variables influencing this path (daily hassles ◊ uncertainty), such as telework (perhaps dog owners work from home more often and it is really working from home that provides that beneficial moderating effect). As such, future studies should consider evaluating the role of the interactions with dogs, rather than merely their presence at home. Furthermore, this result may also be influenced by other contextual or personal variables. For instance, higher median income, personality traits, or even attitudes toward telework may be relevant variables to explore in future studies as control variables or potential moderators.

## Conclusion

This daily-diary study across 10 working days demonstrated that daily hassles increase perceived uncertainty and this, in turn, contributes to adaptive performance; uncertainty may, therefore, signal when employees need to adapt themselves to dynamic daily micro-events. Plus, the presence of a dog during the working day may buffer the detrimental effects of daily hassles on their owners’ perceived uncertainty - pawing effect on employees’ uncertainty. Dogs appear to be the distraction or comfort a person needs when too many daily hassles occur, and uncertainty may threaten the loss of perceived control.

## Data Availability

The data and respective materials are available only upon reasonable request to the author (Ana Junça Silva: ana_luisa_silva@iscte-iul.pt).

## References

[1] Weiss HM, Cropanzano R (1996). Affective events theory: a theoretical discussion of the structure, causes and consequences of affective experiences at work. Res Organizational Behav.

[2] Hecht TD, Cluley H, Lefter AM, Ngamwattana OA. A dynamic framework of boundary permeability: daily events and within-individual fluctuations in daily work and nonwork boundary permeation. *European* Journal of Work and Organizational Psychology; 1–24: 2022.

[3] Junça-Silva A, Mosteo L, Lopes RR (2023). The role of mindfulness on the relationship between daily micro-events and daily gratitude: a within-person analysis. Pers Indiv Differ.

[4] Junça-Silva A, Silva D (2023). The buffering effect of micro-daily events on the relationship between the dark triad traits and counterproductive work behavior. Manage Res Rev.

[5] Anderson EC, Carleton RN, Diefenbach M, Han PK (2019). The relationship between uncertainty and affect. Front Psychol.

[6] Strout TD, Hillen M, Gutheil C, Anderson EC, Hutchinson R, Ward H (2018). Tolerance of uncertainty: a systematic review of health and healthcare-related outcomes. Patient Educ Couns.

[7] Chang W, Atanasov P, Patil S, Mellers BA, Tetlock PE (2017). Accountability and adaptive performance under uncertainty: a long-term view. Judgm Decis Mak.

[8] Hillen MA, Gutheil CM, Strout TD, Smets EM, Han PK. Tolerance of uncertainty: Conceptual analysis, integrative model, and implications for healthcare. Social Science & Medicine, 180; 2017: 62–75. 10.1016/j.socscimed.2017.03.024 28324792

[9] Hesketh B, Neal A, Ilgen DR, Pulakos ED (1999). Technology and performance. The changing nature of performance: implications for staffing, motivation, and development.

[10] Ohly S, Schmitt A (2015). What makes us enthusiastic, angry, feeling at rest or worried? Development and validation of an affective work events taxonomy using concept mapping methodology. J Bus Psychol.

[11] Junça-Silva A (2022). The furr-recovery method: interacting with furry co-workers during work time is a micro-break that recovers workers’ regulatory resources and contributes to their performance. Int J Environ Res Public Health.

[12] Junça-Silva A (2022). Unleashing the Furr-Recovery Method: interacting with Pets in Teleworking replenishes the Self’s Regulatory Resources: evidence from a Daily-Diary Study. Int J Environ Res Public Health.

[13] Etingen, B., Martinez, R. N., Smith, B. M., Hogan, T. P., Miller, L., Saban, K. L.,… Weaver, F. M. Developing an animal-assisted support program for healthcare employees. BMC health services research, 20; 2020: 1–9. 10.1186/s12913-020-05586-8. 10.1186/s12913-020-05586-8 PMC7398398 32746817

[14] Chacko S, Conway N (2019). Employee experiences of HRM through daily affective events and their effects on perceived event-signalled HRM system strength, expectancy perceptions, and daily work engagement. Hum Resource Manage J.

[15] Kelemen TK, Matthews SH, Wan M, Zhang Y (2020). The secret life of pets: the intersection of animals and organizational life. J Organizational Behav.

[16] Chen CY, Hong RY (2010). Intolerance of uncertainty moderates the relation between negative life events and anxiety. Pers Indiv Differ.

[17] Ivancevich JM (1986). Life events and hassles as predictors of health symptoms, job performance, and absenteeism. J Organizational Behav.

[18] Vischer JC (2007). The effects of the physical environment on job performance: towards a theoretical model of workspace stress. Stress and Health: Journal of the International Society for the Investigation of Stress.

[19] Dugas MJ, Gosselin P, Ladouceur R (2001). Intolerance of uncertainty and worry: investigating specificity in a nonclinical sample. Cogn Therapy Res.

[20] Junça-Silva A, Silva D (2022). How is the life without unicorns? A within-individual study on the relationship between uncertainty and mental health indicators: the moderating role of neuroticism. Pers Indiv Differ.

[21] Van den Bos K, Lind EA (2022). Uncertainty management by means of fairness judgments. Res Organizational Behav.

[22] van den Bos K, Lind EA. (2002). Uncertainty management by means of fairness judgments. In M. P. Zanna, editor, *Advances in experimental social psychology*, Vol. 34, pp. 1–60). Academic Press. 10.1016/S0065-2601(02)80003-X.

[23] Freeston MH, Rhéaume J, Letarte H, Dugas MJ, Ladouceur R (1994). Why do people worry?. Pers Indiv Differ.

[24] Greco V, Roger D (2001). Coping with uncertainty: the construction and validation of a new measure. Pers Indiv Differ.

[25] Carleton RN (2016). Into the unknown: a review and synthesis of contemporary models involving uncertainty. J Anxiety Disord.

[26] Yang Q, Van den Bos K, Li Y (2021). Intolerance of uncertainty, future time perspective, and self-control. Pers Indiv Differ.

[27] van den Bos K (2001). Uncertainty management: the influence of uncertainty salience on reactions to perceived procedural fairness. J Personal Soc Psychol.

[28] van den Bos K, Miedema J (2000). Toward understanding why fairness matters: the influence of mortality salience on reactions to procedural fairness. J Personal Soc Psychol.

[29] Taskan B, Junça-Silva A, Caetano A (2022). Clarifying the conceptual map of VUCA: a systematic review. Int J Organizational Anal.

[30] Pulakos ED, Arad S, Donovan MA, Plamondon KE (2000). Adaptability in the workplace: development of taxonomy of adaptive performance. J Appl Psychol.

[31] Pulakos ED, Schmitt N, Dorsey DW, Arad S, Hedge JW, Borman WC (2002). Predicting adaptive performance: further tests of a model of adaptability. Hum Perform.

[32] Pulakos ED, Dorsey DW, White SS, Burke CS, Pierce LG, Salas E (2006). Adaptability in the work place: selecting an adaptive workforce. Understanding adaptability: a prerequisite for effective performance within complex environments.

[33] Hogg MA (2021). Uncertain self in a changing world: a foundation for radicalisation, populism, and autocratic leadership. Eur Rev Social Psychol.

[34] Wagner E, Pina e Cunha M (2021). Dogs at the workplace: a multiple case study. Animals.

[35] Junça-Silva A, Almeida M, Gomes C (2022). The role of dogs in the relationship between telework and performance via affect: a moderated moderated mediation analysis. Animals.

[36] Daltry RM, Mehr KE (2015). Therapy dogs on campus: recommendations for counseling center outreach. J Coll Student Psychother.

[37] Yarborough BJH, Stumbo SP, Yarborough MT, Owen-Smith A, Green CA (2018). Benefits and challenges of using service dogs for veterans with posttraumatic stress disorder. Psychiatr Rehabil J.

[38] MacDonald S. Think like a dog: how Dogs teach us to be happy in life and successful at work. Prestyge Books; 2019.

[39] Delgado-Rodríguez R, Madroñal RC, Villalba CV, Martos-Montes R, Ordoñez-Pérez D (2022). The role of dogs in modulating human affective reactivity and sense of safety in emotional urban public spaces. J Veterinary Behav.

[40] Junça-Silva A. Should I pet or should I work? Human-animal interactions and (tele)work engagement: an exploration of the underlying within-level mechanisms. Personnel Review; 2022. 10.1108/PR-09-2022-0588.

[41] Cunha MPE, Rego A, Munro I (2019). Dogs in organizations. Hum Relat.

[42] Sousa C, Esperança J, Gonçalves G (2022). Pets at work: Effects on social responsibility perception and organizational commitment. Psychol Leaders Leadersh.

[43] Maas CJ, Hox JJ (2005). Sufficient sample sizes for multilevel modeling. Methodology.

[44] Ohly S, Sonnentag S, Niessen C, Zapf D (2010). Diary studies in organizational research. J Personnel Psychol.

[45] Geldhof GJ, Bowers EP, Mueller MK, Napolitano CM, Callina KS, Lerner RM (2014). Longitudinal analysis of a very short measure of positive youth development. J Youth Adolesc.

[46] Junça Silva A, Caetano A, Lopes M. A working day in the life of employees: development and validation of the scale for daily hassles and uplifts at work, 2; 2020: 221–50.

[47] Rafferty AE, Griffin MA (2006). Refining individualized consideration: distinguishing developmental leadershipand supportive leadership. J Occup Organizational Psychol.

[48] Griffin MA, Neal A, Parker SK (2007). A new model of work role performance: positive behavior in uncertain and interdependent contexts. Acad Manag J.

[49] Hox JJ, Boeije HR, Kempf-Leonard K (2005). Data collection, primary vs. secondary. Encyclopedia of Social Measurement.

[50] Rockwood NJ. (2020). MLMED MACRO: Multilevel Mediation in SPSS.

[51] Hayes AF, Rockwood NJ (2017). Regression-based statistical mediation and moderation analysis in clinical research: observations, recommendations, and implementation. Behav Res Ther.

[52] Hox JJ (2010). Multilevel analysis. Techniques and applications.

[53] Junça-Silva A (2023). The Telework Pet Scale: Development and psychometric properties. J Veterinary Behav.

[54] Campbell M, Grimshaw J, Steen N (2000). Changing Professional Practice in Europe Group (EU BIOMED II Concerted Action). Sample size calculations for cluster randomised trials. J Health Serv Res Policy.

[55] Marcoulides GA, Schumacker RE. Advanced structural equation modeling: issues and techniques. Psychology Press; 2013.

[56] Schreiber JB, Nora A, Stage FK, Barlow EA, King J (2006). Reporting structural equation modeling and confirmatory factor analysis results: a review. J Educational Res.

[57] Griep Y, Vanbelle E, Van den Broeck A, De Witte H (2022). Active emotions and personal growth initiative fuel employees’ daily job crafting: a multilevel study. BRQ Bus Res Q.

[58] Smithson M (1999). Conflict aversion: preference for ambiguity vs conflict in sources and evidence. Organ Behav Hum Decis Process.

[59] Park S, Park S (2019). Employee adaptive performance and its antecedents: review and synthesis. Hum Resour Dev Rev.

[60] Sherehiy B, Karwowski W (2014). The relationship between work organization and workforce agility in small manufacturing enterprises. Int J Ind Ergon.

[61] Hall S, Wright H, McCune S, Zulch H (2017). Mills, D. perceptions of dogs in the workplace: the pros and the cons. Anthrozoös.

[62] Wilkin CL, Fairlie P, Ezzedeen SR (2016). Who let the dogs in? A look at pet-friendly workplaces. Int J Workplace Health Manage.

[63] Foreman AM, Glenn MK, Meade BJ, Wirth O (2017). Dogs in the workplace: a review of the benefits and potential challenges. Int J Environ Res Public Health.

[64] Friedmann E. The role of pets in enhancing human well-being: physiological. The Waltham book of human-animal interaction. Benefits and responsibilities of pet ownership; 2013. p. 33.

[65] Gee NR, Mueller MK (2019). A systematic review of research on pet ownership and animal interactions among older adults. Anthrozoös.

[66] Rodriguez KE, Greer J, Yatcilla JK, Beck AM, O’Haire ME, Correction (2021). The effects of assistance dogs on psychosocial health and wellbeing: a systematic literature review. PLoS ONE.

[67] Lundqvist M, Carlsson P, Sjödahl R, Theodorsson E, Levin L. Å. Patient benefit of dog-assisted interventions in health care: a systematic review. BMC complementary and alternative medicine, 17, 1–12; 2017. 10.1186/s12906-017-1844-7 PMC5504801 28693538

[68] Teo JT, Thomas SJ (2019). Psychological mechanisms predicting wellbeing in pet owners: Rogers’ core conditions versus Bowlby’s attachment. Anthrozoös.

[69] Einstein DA (2014). Extension of the transdiagnostic model to focus on intolerance of uncertainty: a review of the literature and implications for treatment. Clin Psychol Sci Pract.

[70] Bordia P, Hunt E, Paulsen N, Tourish D, DiFonzo N (2004). Uncertainty during organizational change: is it all about control?. Eur J work Organizational Psychol.

[71] Bussolari C, Currin-McCulloch J, Packman W, Kogan L, Erdman P (2021). I couldn’t have asked for a better quarantine partner!: experiences with companion dogs during Covid-19. Animals.

[72] Morgan, L., Protopopova, A., Birkler, R. I. D., Itin-Shwartz, B., Sutton, G. A., Gamliel,A., … Raz, T. Human–dog relationships during the COVID-19 pandemic: Booming dog adoption during social isolation. Humanities and Social Sciences Communications, 7(1); 2020.

[73] Podsakoff PM, Podsakoff NP (2019). Experimental designs in management and leadership research: strengths, limitations, and recommendations for improving publishability. Leadersh Q.

[74] Junça Silva A, Neves P, Caetano A (2022). Procrastination is not only a thief of time, but also a thief of happiness: it buffers the beneficial effects of telework on well-being via daily micro-events of IT workers. Int J Manpow.

[75] Schwarzmueller-Erber G, Maier M, Kundi M (2020). Pet attachment and wellbeing of older-aged recreational horseback riders. Int J Environ Res Public Health.

